# Subjective Well-Being in Early Adolescence: Observations from a Five-Year Longitudinal Study

**DOI:** 10.3390/ijerph17218249

**Published:** 2020-11-08

**Authors:** Mònica González-Carrasco, Marc Sáez, Ferran Casas

**Affiliations:** 1 Research Group on Childhood, Adolescence, Children’s Rights and their Quality of Life, Research Institute on Quality of Life, University of Girona, Pujada de Sant Domènec, 9, 17004 Girona, Spain; ferran.casas@udg.edu; 2 Research Group on Statistics, Econometrics and Health. (GRECS), University of Girona and CIBER of Epidemiology and Public Health (CIBERESP), Carrer de la Universitat de Girona 10, Campus de Montilivi, 17003 Girona, Spain

**Keywords:** subjective well-being, late childhood, early adolescence, longitudinal study, gender differences, decreasing-with-age trend, Bayesian models

## Abstract

This article aims to redress the lack of longitudinal studies on adolescents’ subjective well-being (SWB) and highlight the relevance of knowledge deriving from such research in designing public policies for improving their health and wellbeing in accordance with the stage of development they are in. To achieve this, the evolution of SWB during early adolescence (in adolescents aged between 10 and 14 in the first data collection) was explored over a five year period, considering boys and girls together and separately. This involved comparing different SWB scales and contrasting results when considering the year of data collection versus the cohort (year of birth) participants belonged to. The methodology comprised a generalized linear mixed model using the INLA (Integrated Nested Laplace Approximation) estimation within a Bayesian framework. Results support the existence of a decreasing-with-age trend, which has been previously intuited in cross-sectional studies and observed in only a few longitudinal studies and contrasts with the increasing-with-age tendency observed in late adolescence. This decrease is also found to be more pronounced for girls, with relevant differences found between instruments. The decreasing-with-age trend observed when the year of data collection is taken into account is also observed when considering the cohort, but the latter provides additional information. The results obtained suggest that there is a need to continue studying the evolution of SWB in early adolescence with samples from other cultures; this, in turn, will make it possible to establish the extent to which the observed decreasing-with-age trend among early adolescents is influenced by cultural factors.

## 1. Introduction

Subjective well-being (SWB) is among the core concepts within the quality of life paradigm and is considered as part of a positive approach to the study of health, human behaviour, and its determinants. SWB has been conceptualized as how people evaluate their lives, both in general and regarding specific life domains (family, friends, leisure time, etc.) [1]. This definition is applicable not only to adults, but also to children and adolescents [2], for whom SWB is considered to be a very important predictor of different outcomes, including mental health and academic achievement [3].

There is widespread agreement that SWB is composed of three elements, comprising the combination of one cognitive process (satisfaction/dissatisfaction) and two affective processes (positive and negative affect) [4]. The majority of studies focus on either cognitive or emotional aspects, but rarely on both, although some exceptions do exist [5]. The cognitive dimension (specifically, life satisfaction) is considered to be a much more stable component of SWB than the affective component (positive and negative affect) and has been more frequently analysed [3]. 

There is an increasing awareness of the need for children and adolescents’ SWB-based indicators to aid decision making within the context of public policies aimed at this population (see a discussion of this in [6]). Recently, strong emphasis has also been placed on children’s and adolescents’ SWB being considered a product of the intersection between the different contexts in which they are embedded, mainly the family, the neighbourhood, and the school [7], with Bronfenbrenner’s ecological approach [8,9,10] serving as an umbrella in this respect. 

However, the question of whether there are changes in SWB during late childhood and early adolescence has only recently received the attention of researchers working in this field, despite it being a period in which wellbeing would appear to have its own idiosyncrasies with respect to previous and subsequent age groups [11]. Among the reasons for this is that most research has focused on adult samples, in which a relative stability in their levels of SWB has been observed, although with certain oscillations at certain periods of life [12]. This had also led researchers to think that a certain stability would be the predominant pattern during childhood and adolescence, although there are not many studies to support this.

Models that include time as a relevant variable when theorizing on SWB do so in such a generic way that it is difficult to make predictions regarding variations that can occur throughout the life span. Further, most models are not capable of explaining changes in the levels of SWB that may occur at a given time either. As an exception to this, although still formulated with the adult population in mind, we found the homeostatic model [13]. 

This model uses the biological systems of the body that control functions as basic as blood pressure and heart rate as an analogy to explain variations in SWB. Following the example, minimum and maximum values exist for both blood pressure and heart rate that define the expected range of normative levels. Although numerous environmental and internal factors can alter these expected levels (stress and disease, for instance), under normal circumstances, they tend to return to prior values. Based on the above, a homeostatically genetically-driven mechanism is considered to control SWB [13,14]. This would explain the existence of normative values for SWB and relatively small variations for people belonging to the same culture (a standard deviation of only 0.8 points on a 0-100 scale from 26 surveys conducted with Australian adults), except in those cases where the buffers have failed.

The interest of this model in explaining variations in levels of SWB is unquestionable. However, despite expected levels in the aforementioned biological basic functions varying with age (blood pressure tends to increase, while heart rate tends to decrease), this model has not yet incorporated the time variable in a way that helps explain changes occurring in the SWB of children and adolescents, which are quite the opposite of static. This is why it considers the normative values to be the same as for adults [15], even though mean SWB levels have been observed to be higher for most children.

To this, we must add the fact that the majority of studies on children’s and adolescents’ SWB are of a cross-sectional nature, which acts as a hindrance to gaining in-depth knowledge on how the evolutionary changes that take place throughout their development affect or are affected by their SWB. Some years ago, various cross-sectional studies started revealing that this supposed stability might not be taking place [11], a result that was later confirmed when scales with more response options were included (0–10-point scales) and particularly, when involving longitudinal studies. Two main results were observed. On the one hand, a decreasing-with-age trend, according to which levels of SWB decrease from 10 years of age onwards [5,16], although some studies situate this decrease between 13 and 15 (Shek & Liu, cited in [3]). On the other hand, that the pattern of decline is more pronounced among girls than boys, the former obtaining lower levels than the latter despite starting from higher scores [16]. It has also been observed that factors contributing to a decrease in SWB levels differ from those contributing to its increase and are gender sensitive [17]. 

However, some contradictory results have been observed in reference to late adolescence when it comes to gender. For instance, Steinmayr et al. [3] did not observe any association between gender and satisfaction with life at any of the four data collection points throughout the three years of the longitudinal study they performed with late German adolescents (mean of 16.43 at the beginning of the study). In a study of Finnish samples, which ranged from 642 to 749 cases depending on the time of data collection, Salmela-Aro and Tuominen-Soini [12] found that life satisfaction increased among 15 to 17-year-old girls (and also among boys, although they displayed more homogenous pathways than girls) during the transition from comprehensive school to their subsequent educational pathway.

Knowing exactly when this decrease takes place and what can cause it will allow for actions to be introduced for its prevention, especially in those cases where it might be particularly pronounced and in which external environment factors can play a very important role. Another noteworthy observation is that longitudinal studies have frequently used only single-item life satisfaction measures (Lucas & Donnellan, cited in [12]), despite different instruments for measuring SWB appearing to be sensitive to detecting these changes to a greater or lesser degree and multiple-item SWB scales being more sensitive (i.e., they take into account the measurement of different life domains when evaluating SWB) than single-item scales [16]. However, these results were observed with only a two year follow-up study. This would need to be lengthened to determine the extent to which these first observations remain valid with a longer follow up—precisely one of the aims of this article.

A relevant issue that has not yet been sufficiently explored is which indicator best monitors the evolution of SWB over time: year of birth (generally referred to as cohort), year of data collection, or both at the same time. This debate cannot be separated from that regarding which statistical technique is most appropriate for studying this evolution. Since SWB does not follow a Gaussian distribution due to the existence of an “optimistic bias” (the mean is not at the midpoint, but shifted to the right on a scale from less to more), many statistical tests have important limitations in this regard. 

Taking into account all of the above, the aim of this article is to explore the evolution of early adolescents’ SWB over a five year period, considering boys and girls together and separately. This involves comparing different SWB scales and results when considering the year of data collection versus the cohort participants belonged to. To achieve this, a generalized linear mixed model will be constructed using the INLA (Integrated Nested Laplace Approximation) estimation within a Bayesian framework, as described in [18,19]. 

## 2. Materials and Methods

### 2.1. Participants

Data were collected from an overall sample of 2180 Catalan adolescents (i.e., from Catalonia, north-east Spain) over a period of five consecutive years. Of these adolescents, 484 only responded once, 755 twice, 539 three times, 213 responded for four consecutive years, and 189 answered for five consecutive years (Table 1). In order to allow detailed analysis of the differences between data collections and cohorts, data from participants who only answered the questionnaire once were retained.

Data collection included nine cohorts, born between 1998 and 2006. Those that answered five times were born between 1999 and 2002. All cohorts included slightly more girls than boys, except for 2001 (Table 2). In the first year of data collection, most participants were aged between 10 and 14—and therefore, belong to the category of early adolescence [20] with some, though only few, 9 and 16-year-olds. The following years, new cohorts of 10–11 year-olds were added. By the fifth year of data collection, those aged 10 in the first year were 14 years old, those aged 11 were 15, and so on.

### 2.2. Instruments

Following suggestions by Casas [6], several instruments have been used in order to compare the results obtained by each of them, the chosen scales being among the most widely used according to the age of the participants in the study. Scales for measuring SWB can be classified into the following categories [21]: (1) Single item scales (which evaluate SWB using only one item, formulated in a generic way), (2) Multiple item context free scales (which evaluate SWB using multiple items, but referring to the same construct), and (3) Multiple item domain based scales (including the measurement of different life domains when evaluating SWB). All of the instruments were administered in the Catalan language since this is the vehicular language at schools in Catalonia. They have also been used in previous studies after back-translation from English and piloting with children and adolescents of different ages.

#### 2.2.1. Single Item Scales of Subjective Wellbeing

##### Overall Life Satisfaction (OLS)

The importance of including a single item scale on overall satisfaction when studying SWB has been highlighted by Campbell et al. [1]. In our research, we included a question on satisfaction with your overall life, using an end-labelled 0–10 scale, from completely dissatisfied to completely satisfied.

##### Happiness Taking Into Account Overall Life (HOL)

Campbell et al. [1] also pointed out the importance of including a single item scale on happiness when studying SWB. In our research, we have included the following question: “Taking into account your overall life, would you say you are…?” and options are then offered using a 0–10 scale, from extremely unhappy to extremely happy.

#### 2.2.2. Multiple Item Context Free Scale of Subjective Wellbeing

##### Satisfaction with Life Scale (SWLS) and Student’s Life Satisfaction Scale (SLSS)

The five original items of the SWLS scale [22] were used for all children during the first two years of data collection. From the third year on, we decided to use the SLSS modified version from the Children’s Worlds project ([23]; www.isciweb.org)—based on Huebner’s [24] SLSS scale—for the youngest children in order to improve understanding and data quality. They are both context free, multi item scales with equivalent items, with adapted wording for younger children in the case of the SLSS.

The SLSS includes five items; responses were originally encoded on a scale of 1–7 according to level of agreement. The version adapted by Casas et al. [25] was used in this research, with the 1–7 scale changed to a 0–10 scale in order to make it more sensitive and only four of the items used for data analysis, with Item 5 being excluded for the reasons explained in Casas et al. [25]. The labels Strongly agree and Strongly disagree were only used for the end values 0 and 10.

#### 2.2.3. Multiple Item Domain Based Subjective Wellbeing Scales

##### Personal Well-Being Index (PWI)

This scale was designed as part of the Australian Unity Wellbeing Index. It originally included seven items on satisfaction with different life domains. Although the PWI was designed for use with adults, it has been tested on 12 year-olds and older adolescents in some countries, showing good psychometric properties [25,26]. In this research, we applied the adult version (PWI-A) to the second year of the 2nd cohort (mostly 12 year-olds) and subsequent cohorts. The seven items explored are: satisfaction with your standard of living, with your health, with what you are achieving in life, with feeling part of the community, with your personal relationships, with how safe you feel, and with your future security. The scale has an end-labelled format, from completely dissatisfied (0) to completely satisfied (10). The item referring to “satisfaction with feeling part of the community” has been substituted by “satisfaction with the groups of people I belong to”, as in previous Spanish samples, explained in Casas et al. [25,26].

Because the longitudinal study involved participants of different ages and therefore with different cognitive skills, a schoolchildren’s version of the PWI (PWI-SC), developed by Cummins and Lau [27], was applied to the 1st cohort (mostly 10 year-olds) and the first year of the 2nd cohort (mostly 11 year-olds). The seven items on this index are as follows: satisfaction about the things you have, with your health, with the things you want to be good at, about how safe you feel, about doing things away from your home, and about what may happen to you later on in your life. The scale has an end-labelled format, from completely dissatisfied (0) to completely satisfied (10). In order to improve participants’ comprehension, three emoticons corresponding to the values 0, 5, and 10 were included in this version, in line with the findings from a pilot study conducted previously [28].

##### Brief Multidimensional Student’s Life Satisfaction Scale (BMSLSS)

This scale was developed to be used with students aged 8–18. It includes five items referring to satisfaction with different life domains plus one item on overall satisfaction. The psychometric properties of this scale have been published in different articles [29,30,31]. Responses were originally encoded on a scale of 1–7, from terrible to delighted. The 1–7 scale was changed to a 0–10 scale in order to make it more sensitive. Labels were given to each value, describing satisfaction with each life domain from terrible to delighted.

### 2.3. Procedure and ethics

After the Department of Education had authorized the research, the schools willing to participate in the study were asked about the most convenient dates for administering the questionnaire. The main reason for some schools not wishing to participate was that a longitudinal study requires commitment over several years, requiring their participation in many data collections, hence the sampling being non-probabilistic.

When the research was initiated, approval from the Ethics Committee was not compulsory at the university to which the authors belong. Consent was previously requested from the parents or legal guardians of the students involved in the study. Once consent was obtained, paper questionnaires were administered in the classroom setting in consecutive years. Researchers were present during administration in order to answer questions and give instructions on how to complete the questionnaires. The regular teacher was also present to help the researchers organize the classroom.

Participants were notified that confidential treatment of the information they provided was explicitly guaranteed. All participants were informed that they were free to refuse participation and not to answer any of the questions if they accepted to participate.

### 2.4. Data Analysis

Linear mixed models with variable response from the Gaussian family were used. The following were included as explanatory variables in the model: gender, year of response to the questionnaire, and cohort (year of birth: 1998–2006). A random effect was also included in the model to capture individual heterogeneity; this comprised unobserved confounders specific to each child and invariant in time that in addition to sex, year, and cohort, could explain the response variable.

The relationships between both the response (or dependent) variables and the year and the cohort were allowed to be non-linear. Inferences were made following a Bayesian perspective, by means of the INLA approach [32,33]. Priors that penalize complexity (called PC priors) were used. These priors are robust in the sense that they do not have an impact on the results and in addition, have an epidemiological interpretation [34]. All analyses were carried out using the free software R (version 4.0.0) (The R Foundation for Statistical Computing, Vienna, Austria) [35,36], through the INLA package [31,32,35].

## 3. Results

### 3.1. Single Item Scales of Subjective Wellbeing

#### Overall Life Satisfaction (OLS)

There was a statistically significant reduction in the OLS scores across the five year period considered; that is, comparing the first and fifth data collection, this reduction is significantly higher among girls (1.18 points) (Table 3).

The reduction in the mean was significant for all data collections, regardless of gender (Table 4).

**Figure 1:**
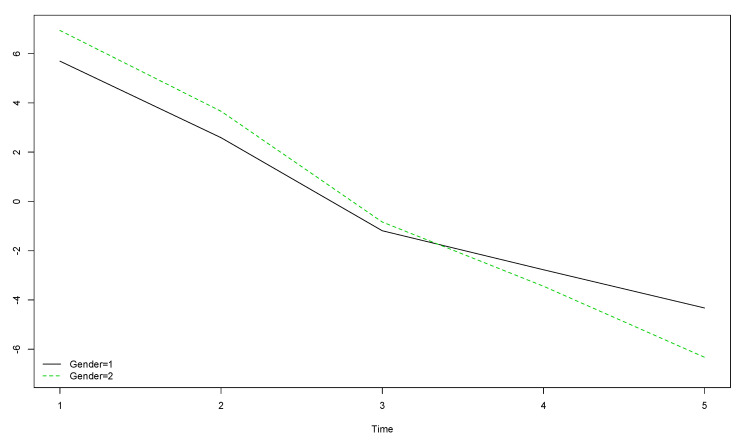
Boys’ and girls’ trends for OLS scores over the five year longitudinal study by time of data collection (the vertical axis represents the original scores obtained with the instrument).

The comparison between boys’ and girls’ OLS scores when year of data collection is considered showed that girls’ (gender = 2) OLS scores were higher than boys’ (gender = 1) at the start of the longitudinal study, but lower by the end, meaning this decrease was more pronounced among girls (Figure 1). 

**Figure 2:**
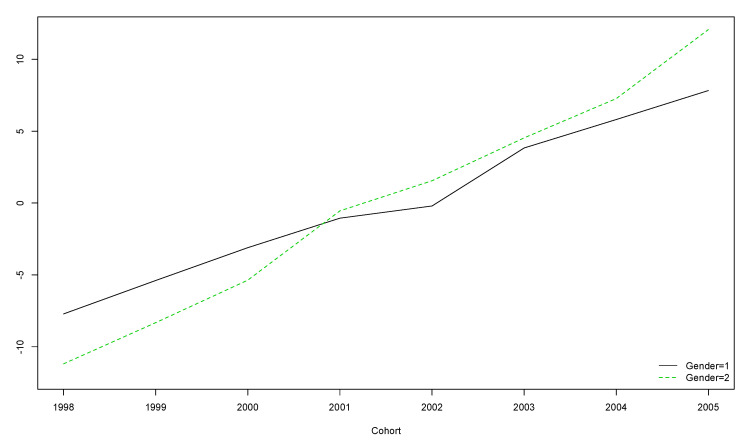
Boys’ and girls’ trends for OLS scores over the five year longitudinal study by cohort (the vertical axis represents the original scores obtained with the instrument).

A decreasing-with-age trend was observed regardless of gender (Figure 2). However, for boys born in the years 2001 and 2002 and girls born in 2001, this trend was not significant (Table 5).

The older the participants, the lower their score on the OLS, regardless of gender (Figure 2).

#### Happiness Overall (HOL)

There was a statistically significant reduction in HOL scores across the five year period considered; that is, comparing the first and fifth data collection, this reduction is significantly higher among girls (1.46 points) (Table 6).

The reduction in the mean was significant for all data collections, regardless of gender (Table 7).

**Figure 3:**
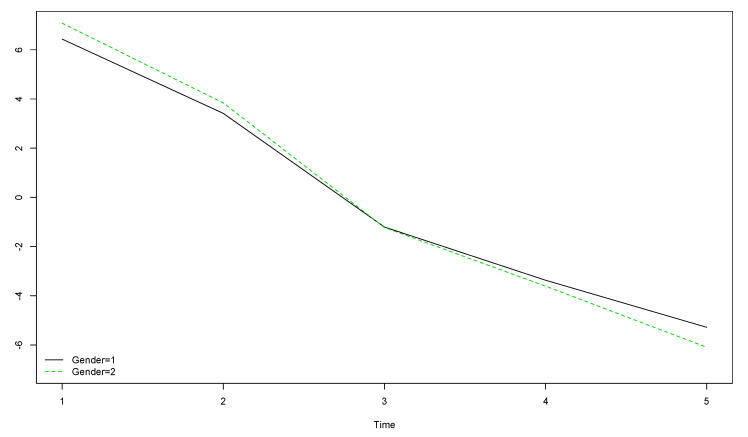
Boys’ and girls’ trends for HOL scores over the five year longitudinal study by time of data collection (the vertical axis represents the original scores obtained with the instrument).

Girls’ (gender = 2) HOL scores were slightly higher than boys’ (gender = 1) at the start of the longitudinal study and slightly lower by the end (Figure 3). Differences between boys and girls were smaller for the HOL compared to the PWI and the OLS.

The decreasing-with-age trend was observed for all cohorts, with the exception of boys and girls born in the years 2001 and 2002 (Table 8).

**Figure 4:**
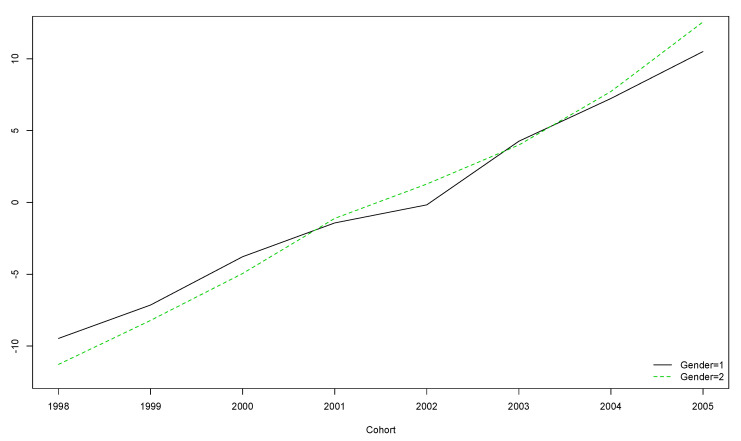
Boys’ and girls’ trends for HOL scores over the five year longitudinal study by cohort (the vertical axis represents the original scores obtained with the instrument).

The older the participants, the lower their score, regardless of gender (Figure 4).

### 3.2. Multiple Item Context Free Scale of Subjective Wellbeing

#### Satisfaction with Life Scale (SWLS) and Student’s Life Satisfaction Scale (SLSS)

There was a statistically significant reduction in SWLS/SLSS scores across the five year period considered; that is, comparing the first and fifth data collection, this reduction is significantly higher among girls (4.57 points) (Table 9).

The reduction in the mean was significant for all data collections for both boys and girls (Table 10).

**Figure 5:**
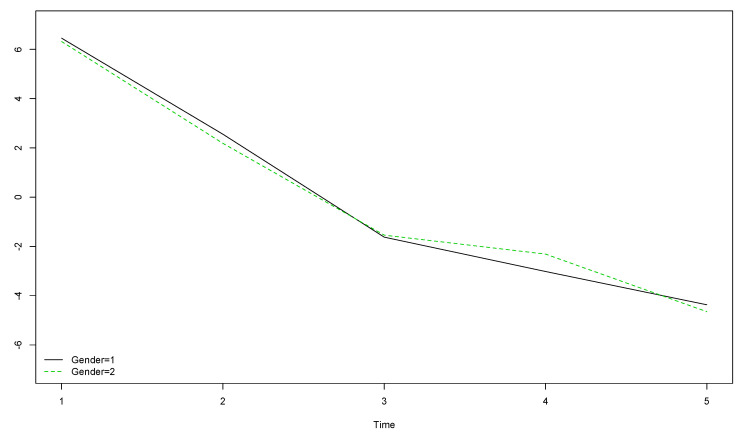
Boys’ and girls’ trends for SWLS/SLSS scores over the five years longitudinal study by time of data collection (the vertical axis represents the original scores obtained with the instrument).

Girls’ (gender = 2) SWLS/SLSS scores were the same as boys’ (gender = 1) at the start of the longitudinal study and almost the same at the end (Figure 5).

The decreasing-with-age trend was observed for all cohorts, with the exception of boys and girls born in the years 2001 and 2002 (Table 11).

**Figure 6:**
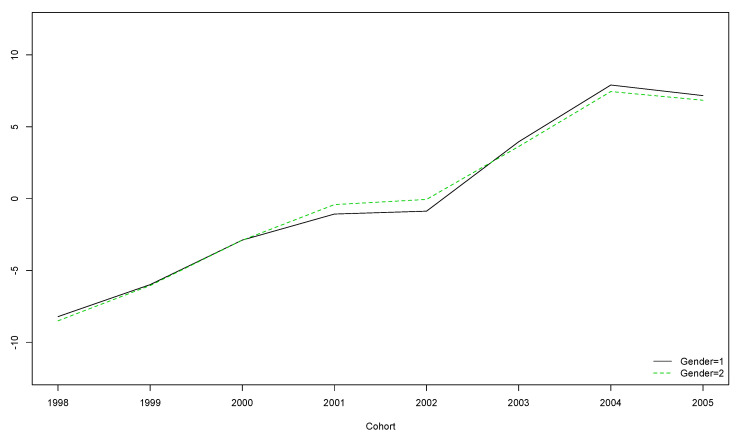
Boys’ and girls’ trends for SWLS/SLSS scores over the five year longitudinal study by cohort (the vertical axis represents the original scores obtained with the instrument).

The older the participants, the lower the mean score, regardless of gender (Figure 6).

### 3.3. Multiple Item Domain Based Subjective Wellbeing Scale

#### Personal Wellbeing Index (PWI)

There was a statistically significant reduction in the PWI scores (almost 1 point on a 0–11 point scale departing from a mean of 85.55) across the five year period considered; that is, comparing the first and fifth data collection, this reduction is significantly higher among girls (1.23 points) (Table 12).

When the year of data collection is taken into account, we observed that boys from the third year of data collection fell outside this trend as the reduction in the mean was not significant. For the rest of the years of data collection, this decrease in the PWI mean was statistically significant (Table 13). In the case of girls, this decrease was statistically significant for all years when data were collected.

**Figure 7:**
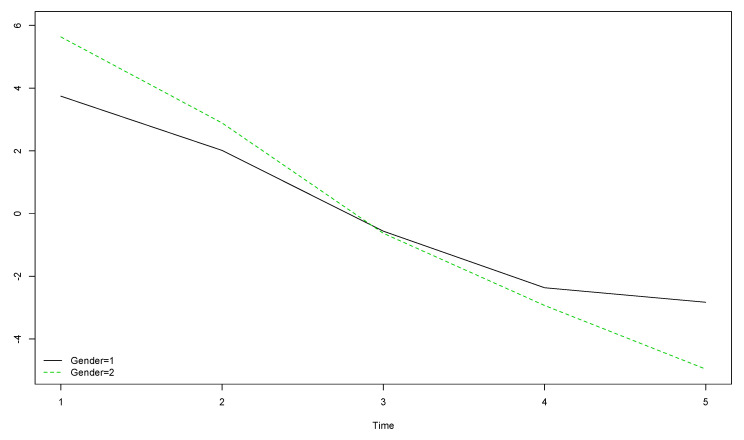
Boys’ and girls’ trends for PWI scores over the five year longitudinal study by time of data collection (the vertical axis represents the original scores obtained with the instrument).

The comparison of boys’ and girls’ PWI scores showed that girls’ (gender = 2) scores were higher than boys’ (gender = 1) at the start of the longitudinal study but lower at the end, meaning this decrease was more pronounced for girls (Figure 7).

The decreasing-with-age trend observed was significant for all cohorts, with the exception of both boys and girls born in the years 2001 and 2002 (Table 14).

**Figure 8:**
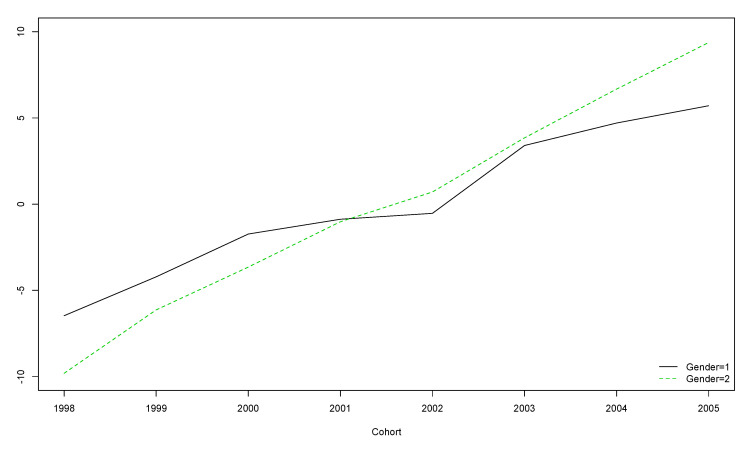
Boys’ and girls’ trends for PWI scores over the five year longitudinal study by cohort (the vertical axis represents the original scores obtained with the instrument).

The older the participants, the lower the PWI scores, regardless of gender (Figure 8).

#### Brief Multidimensional Student’s Life Satisfaction Scale (BMSLSS)

There is a statistically significant reduction in BMSLSS scores across the five year period considered; that is, comparing the first and fifth data collection, this reduction is significantly higher among girls (1.75 points) (Table 15).

The reduction in the mean is significant for all data collections (Table 16), regardless of gender.

**Figure 9:**
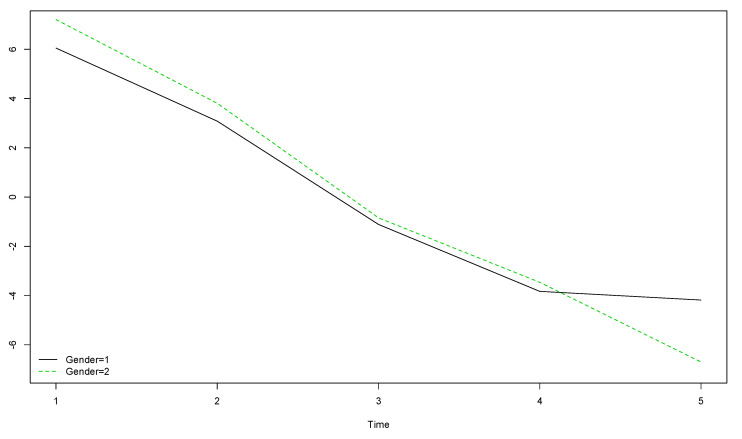
Boys’ and girls’ trends for BMSLSS scores over the five years longitudinal study by time of data collection (the vertical axis represents the original scores obtained with the instrument).

Girls’ (gender = 2) BMSLSS scores were higher than boys’ (gender = 1) at the start of the longitudinal study, but lower at the end. This decrease was more pronounced for girls. For boys, the trend in BMSLSS scores stabilizes between the fourth and fifth years of data collection (Figure 9).

The decreasing-with-age trend described is observed for all cohorts, with the exception of boys born in 2002 and girls born in 2001 (Table 17).

**Figure 10:**
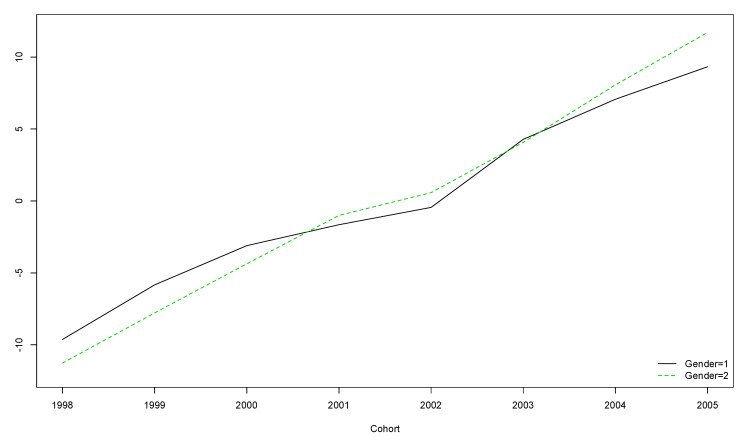
Boys’ and girls’ trends for BMSLSS scores over the five years longitudinal study by cohort (the vertical axis represents the original scores obtained with the instrument).

Boys and girls born in 1998 have lower mean scores than participants from other cohorts and those born in 2005 have the highest mean scores (Figure 10).

## 4. Discussion

The global means (considering all five data collections at the same time) for the five instruments used to evaluate SWB differ by 7.9 points on a 0–100 scale (from 78.33 for the SWLS/SLSS to 86.23 for the OLS). Although these differences are not very high, the fact that they exist raises the question of whether they are measuring exactly the same concept, as has already been highlighted by Casas [6], which would make it advisable not to limit data collection to only one of them. For all instruments, the means are above the normative values defined for Western adult populations (mean of 75 on a 0–100 scale, Cummins [14]), although the mean is only slightly above this for the SWLS/SLSS. This does not imply that the homeostatic model is not equally applicable to the non-adult population, but simply that normative values for adolescents should be revised, as opposed to that argued by Cummins [15]. Because instruments are generally used either to assess the cognitive or affective dimensions of SWB, in this study, two instruments of the same type (single item scales) that assess the cognitive (the OLS) and affective dimensions (the HOL), respectively, were applied. The means obtained with the two instruments are similar (86.23 versus 85.91).

The results obtained support the decreasing-with-age trend previously intuited in cross-sectional studies and clearly observed in a Spanish longitudinal study starting with 10-year-olds [5,16] and in a 6-year longitudinal study with a large sample of Chinese adolescents (N = 3.328) starting with 12-year-olds [37]. This statistically significant reduction in levels of SWB with age, taking the global mean, is observed regardless of the instrument used. Thus, it is observed in the case of single item scales (the OLS and HOL), a multiple item context free scale (the SWLS/SLSS), and multiple item domain based scales (the PWI and BMSLSS). It is worth mentioning that the decreasing-with-age tendency is opposite to that found with samples of late adolescents (16 to 19 year-olds), for whom an increase in SWB is detected (see the work by Steinmayr et al. [3]). This reinforces the importance of conducting in-depth research into the period before puberty and the moment when puberty takes place (around age 10: Landsford & Banati [20]) as they differ from earlier and later stages of adolescence.

However, these results were obtained from cross-sectional studies (longitudinal comparative studies with samples from different socio-cultural contexts are also necessary), suggesting that there are differences in the age at which the decreasing-with-age tendency stabilizes. There is therefore a need for more studies with children under 12 in order to further our knowledge regarding the age at which this tendency starts, even if it is suspected that there are also cultural variations in this regard (see [37]).

This decrease is also found to be more pronounced for girls, with relevant differences found between instruments, especially with regard to the SWLS/SLSS (a decrease of 4.57 points on a 100-point scale) compared to the rest (between 1.18 for the OLS and 1.75 for the BMSLSS). This differs from the study conducted by Steinmayr et al. [3], who did not observe any association between gender and satisfaction with life at any of the four data collection points throughout the three years of the longitudinal study they performed with late German adolescents (mean of 16.43 at the beginning of the study). It even contradicts the results obtained by Salmela-Aro and Tuominen-Soini [12] who found that life satisfaction increased among 15 to 17-year-olds girls (and also among boys, although they displayed more homogenous pathways than girls) during the transition from comprehensive school to their subsequent educational pathway. The authors hypothesized that this increase might be the result of a better fit with their school environment (they can choose what to continue studying at this age).

However, it should be noted that in both studies, participants were late adolescents, not early adolescents as in this study. Another explanation may be found in social role theory, according to which boys’ and girls’ behaviours differ because above and beyond their physical differences, they have been attributed different roles in society [38]). Thus, the more different the roles they are attributed, the more differences there are expected to be in terms of SWB pathways.

When the year of data collection is taken into account, interesting differences are observed among instruments. In the case of the OLS, the PWI, and the BMSLSS, the decrease is clearly higher among girls across the five year period considered, despite their initial scores being higher than boys’. In the case of the BMSLSS, the decreasing-with-age trend stabilizes for boys from the fourth to the fifth year of data collection, a result that was not observed with any of the other four instruments. With regard to the HOL, differences between girls and boys are much smaller and almost non-existent when measured using the SWLS/SLSS. This again indicates not only the suitability of using different types of instruments to explore SWB at these ages, as pointed out by Casas [6] and also done in Casas and González-Carrasco [39], but also that the available instruments seem to be more gender-sensitive than may have been thought.

The decreasing-with-age trend previously observed when the year of data collection was taken into account is also observed when considering the cohort, but the latter provides additional information. Specifically, there are exceptions to this trend for some cohorts, such as those born in the years 2001 and 2002, for both girls and boys. This applies to all instruments except the OLS, for which this exception only applies for girls born in 2001. These results suggest that despite the trend observed for the cohort and the year of data collection being the same, nuances exist that would not have been observed if the two variables had not been considered at the same time. The aim of this article was to identify variations in SWB in early adolescence by age and cohort. For future studies, the challenge remains to delve deeper into the factors that contribute to such variations, something that was beyond the scope of this study.

## 5. Conclusions

Thanks to the longitudinal design adopted here, the study has given support to the existence of a decreasing-with-age trend in SWB scores within the period of early adolescence, a trend that previous authors have considered to be a “developmental phenomenon” (Goldbeck et al. [40], p. 969). It contrasts with an increasing-with-age trend observed among late adolescence in some other longitudinal studies. Knowing at what age this decline occurs and the factors that cause it is very important in order to carry out preventive actions that reduce any negative consequences that may be associated with this decrease. Further, for this, longitudinal studies are essential. The fact that the pattern of decrease is different for boys and girls suggests it is worth considering targeting different actions at different times.

Another consequence deriving from the results obtained here is the need to carry out longitudinal studies with more culturally diverse and wider samples than the one used here in order to establish the role that culture has on the age of onset of this decline. This article therefore constitutes just one example of the huge cultural diversity that potentially exists among early adolescents’ SWB trajectories and opens the door to continue investigating these issues in greater depth. In order to do this, it would also be necessary to continue comparing the results obtained with different instruments that cover both the affective and cognitive dimensions of SWB, while also including a complementary construct, that of psychological well-being (PWB).

## Figures and Tables

**Table 1:** Sample characteristics.

Data Collections and Cohorts		Number of Years the Child Answered the Questionnaire					Total
		1	2	3	4	5	
Cohort (year of birth)	1998	16	134	40	39	0	229
	1999	25	82	69	22	13	211
	2000	28	86	51	86	39	290
	2001	18	88	27	31	69	233
	2002	11	76	61	35	68	251
	2003	77	112	136	0	0	325
	2004	29	46	132	0	0	207
	2005	57	131	23	0	0	211
	2006	223	0	0	0	0	223
Total		484	755	539	213	189	2180

**Table 2:** Number of years the child answered the questionnaire by cohort and gender.

Cohort and Gender		Cohort										Total
		1998	1999		2000	2001	2002	2003	2004	2005	2006	
boy	Number of years the child answered the questionnaire	1	8	9	15	9	5	39	11	26	99	221
		2	59	39	45	49	37	52	19	59	0	359
		3	21	25	19	19	35	68	54	8	0	249
		4	17	10	35	14	9	0	0	0	0	85
		5	0	2	13	40	32	0	0	0	0	87
	Total	105	85		127	131	118	159	84	93	99	1001
girl	Number of years the child answered the questionnaire	1	8	16	13	9	6	38	18	31	124	262
		2	75	43	41	39	39	60	27	72	0	396
		3	19	44	32	8	26	68	78	15	0	290
		4	22	12	51	17	26	0	0	0	0	128
		5	0	11	26	29	36	0	0	0	0	102
	Total	124	126		163	102	133	166	123	118	124	1179
Total	Number of years the child answered the questionnaire	1	16	25	28	18	11	77	29	57	223	483
		2	134	82	86	88	76	112	46	131	0	755
		3	40	69	51	27	61	136	132	23	0	539
		4	39	22	86	31	35	0	0	0	0	213
		5	0	13	39	69	68	0	0	0	0	189
	Total	229	211		290	233	251	325	207	211	223	2180

**Table 3:** Variations in mean Overall Life Satisfaction (OLS) scores over the five year longitudinal study.

	Coefficients	0.025 Quantile	0.975 Quantile	Probability of Coefficient Differing from Zero
Intercept	86.23	85.46	87.00	1.00
gender (girls)	−1.18	−2.22	−0.14	0.99

**Table 4:** Variations in mean OLS scores by time of data collection.

Gender	Year of Data Collection	M	*SD*	0.025 Quantile	0.975 Quantile
Boys	1st	5.70	0.51	4.69	6.70
	2nd	2.59	0.48	1.65	3.53
	3rd	−1.19	0.42	−2.00	−0.37
	4th	−2.77	0.45	−3.66	−1.88
	5th	−4.32	0.51	−5.32	−3.32
Girls	1st	6.94	0.47	6.03	7.86
	2nd	3.66	0.44	2.79	4.53
	3rd	−0.83	0.39	−1.59	−0.07
	4th	−3.44	0.43	−4.28	−2.60
	5th	−6.33	0.48	−7.27	−5.38

**Table 5:** Variations in mean OLS scores by cohort.

Gender	Cohort	M	*SD*	0.025 Quantile	0.975 Quantile
Boys	1998	−7.71	0.97	−9.62	−5.81
	1999	−5.39	0.95	−7.26	−3.52
	2000	−3.10	0.82	−4.72	−1.49
	2001	−1.059	0.79	−2.61	0.50
	2002	−0.20	0.81	−1.79	1.38
	2003	3.83	0.83	2.20	5.46
	2004	5.81	0.96	3.93	7.69
	2005	7.83	1.13	5.62	10.04
Girls	1998	−11.19	0.90	−12.97	−9.41
	1999	−8.32	0.84	−9.97	−6.68
	2000	−5.37	0.73	−6.80	−3.94
	2001	−0.54	0.85	−2.21	1.11
	2002	1.55	0.76	0.05	3.04
	2003	4.53	0.81	2.95	6.11
	2004	7.27	0.85	5.59	8.94
	2005	12.07	1.02	10.06	14.08

**Table 6:** Variations in mean Happiness Taking into Account Overall Life (HOL) scores over the five year longitudinal study.

	Coefficients	0.025 Quantile	0.975 Quantile	Probability of Coefficient Different from Zero
intercept	85.91	85.14	86.69	1.00
gender (girls)	−1.46	−2.50	−0.41	0.99

**Table 7:** Variations in mean HOL scores by time of data collection.

Gender	Year of Data Collection	M	*SD*	0.025 Quantile	0.975 Quantile
Boys	1st	6.43	0.48	5.481	7.35
	2nd	3.42	0.44	2.52	4.27
	3rd	−1.20	0.38	−1.95	−0.452
	4th	−3.37	0.41	−4.18	−2.55
	5th	−5.28	0.48	−6.21	−4.31
Girls	1st	7.08	0.43	6.24	7.95
	2nd	3.84	0.41	3.04	4.66
	3rd	−1.22	0.35	−1.91	−0.56
	4th	−3.61	0.39	−4.38	−2.83
	5th	−6.09	0.45	−7.00	−5.21

**Table 8:** Variations in mean HOL scores by cohort.

Gender	Cohort	M	*SD*	0.025 Quantile	0.975 Quantile
Boys	1998	−9.47	0.96	−11.34	−7.56
	1999	−7.14	0.93	−8.97	−5.31
	2000	−3.781	0.82	−5.35	−2.13
	2001	−1.43	0.78	−2.97	0.09
	2002	−0.17	0.84	−1.87	1.42
	2003	4.26	0.83	2.67	5.94
	2004	7.24	0.93	5.41	9.08
	2005	10.51	1.15	8.20	12.70
Girls	1998	−11.28	0.90	−13.08	−9.54
	1999	−8.22	0.83	−9.85	−6.59
	2000	−4.95	0.73	−6.41	−3.53
	2001	−1.11	0.83	−2.72	0.53
	2002	1.28	0.79	−0.22	2.86
	2003	3.40	0.81	2.37	5.55
	2004	7.72	0.84	6.06	9.37
	2005	12.57	1.04	10.56	14.64

**Table 9:** Variations in mean SWLS/SLSS scores over the five year longitudinal study.

	Coefficients	0.025 Quantile	0.975 Quantile	Probability of Coefficient Differing from Zero
Intercept	78.33	76.87	79.79	1.00
gender (girls)	−4.57	−6.27	−2.87	0.99

**Table 10:** Variations in mean SWLS/SLSS scores by time of data collection.

Gender	Year of Data Collection	M	SD	0.025 Quantile	0.975 Quantile
Boys	1st	6.45	0.71	5.06	7.85
	2nd	2.55	0.58	1.41	3.70
	3rd	−1.62	0.53	−2.67	−0.58
	4th	−3.02	0.58	−4.16	−1.87
	5th	−4.37	0.68	−5.71	−3.03
Girls	1st	6.32	0.62	5.09	7.54
	2nd	2.18	0.54	1.11	3.25
	3rd	−1.54	0.50	−2.53	−0.55
	4th	−2.31	0.56	−3.41	−1.21
	5th	−4.65	0.65	−5.93	−3.37

**Table 11:** Variations in mean SWLS/SLSS scores by cohort.

Gender	Cohort	M	SD	0.025 Quantile	0.975 Quantile
Boys	1998	−8.21	1.28	−10.73	−5.70
	1999	−5.98	1.21	−8.36	−3.61
	2000	−2.88	1.07	−4.99	−0.78
	2001	−1.07	1.06	−3.15	1.00
	2002	−0.87	1.11	−3.06	1.313
	2003	3.96	1.05	1.88	6.03
	2004	7.90	1.32	5.31	10.50
	2005	7.17	2.70	1.86	12.47
Girls	1998	−8.50	1.22	−10.90	−6.11
	1999	−6.05	1.13	−8.27	−3.83
	2000	−2.89	1.00	−4.87	−0.92
	2001	−0.42	1.08	−2.54	1.70
	2002	−0.06	1.09	−2.19	2.08
	2003	3.63	1.04	1.59	5.66
	2004	7.45	1.24	5.01	9.89
	2005	6.84	2.66	1.61	12.07

**Table 12:** Variations in mean Personal Well-Being Index (PWI) scores over the five year longitudinal study.

	Coefficients	0.025 Quantile	0.975 Quantile	Probability of Coefficient Differing from Zero
intercept	85.55	84.74	86.35	1.00
gender (girls)	−1.23	−2.01	−0.44	0.99

**Table 13:** Variations in mean PWI scores by time of data collection.

Gender	Year of Data Collection	M	*SD*	0.025 Quantile	0.975 Quantile
Boys	1st	3.74	0.40	2.96	4.52
	2nd	2.01	0.35	1.32	2.70
	3rd	−0.56	0.30	−1.15	0.026
	4th	−2.36	0.33	−3.02	−1.71
	5th	−2.83	0.40	−3.61	−2.04
Girls	1st	5.63	0.36	4.93	6.33
	2nd	2.89	0.32	2.25	3.52
	3rd	−0.63	0.27	−1.17	−0.093
	4th	−2.93	0.31	−3.54	−2.32
	5th	−4.96	0.38	−5.71	−4.21

**Table 14:** Variations in mean PWI scores by cohort.

Gender	Cohort	M	*SD*	0.025 Quantile	0.975 Quantile
Boys	1998	−6.47	0.78	−8.00	−4.92
	1999	−4.21	0.76	−5.72	−2.73
	2000	−1.73	0.66	−3.01	−0.41
	2001	−0.87	0.61	−2.07	0.31
	2002	−0.53	0.63	−1.79	0.69
	2003	3.40	0.65	2.15	4.70
	2004	4.71	0.76	3.20	6.21
	2005	5.71	0.96	3.81	7.58
Girls	1998	−9.81	0.74	−11.28	−8.36
	1999	−6.13	0.67	−7.46	−4.80
	2000	−3.65	0.59	−4.83	−2.49
	2001	−1.02	0.65	−2.29	0.26
	2002	0.71	0.59	−0.44	1.88
	2003	3.85	0.62	2.61	5.06
	2004	6.67	0.68	5.33	8.03
	2005	9.38	0.88	7.66	11.13

**Table 15:** Variations in mean Brief Multidimensional Student’s Life Satisfaction Scale (BMSLSS) scores over the five years longitudinal study.

	Coefficients	0.025 Quantile	0.975 Quantile	Probability of Coefficient Differing from Zero
intercept	84.92	84.28	85.55	1.00
gender (girls)	−1.75	−2.60	−0.89	0.99

**Table 16:** Variations in mean BMSLSS scores by time of data collection.

Gender	Year of Data Collection	M	SD	0.025 Quantile	0.975 Quantile
Boys	1st	6.04	0.37	5.32	6.77
	2nd	3.08	0.36	2.37	3.79
	3rd	−1.11	0.31	−1.72	−0.50
	4th	−3.83	0.34	−4.50	−3.16
	5th	−4.18	0.38	−4.92	−3.44
Girls	1st	7.21	0.33	6.55	7.86
	2nd	3.80	0.32	3.17	4.44
	3rd	−0.85	0.28	−1.40	−0.29
	4th	−3.46	0.32	−4.08	−2.84
	5th	−6.70	0.35	−7.39	−6.01

**Table 17:** Variations in mean BMSLSS scores by cohort.

Gender	Cohort	M	SD	0.025 Quantile	0.975 Quantile
Boys	1998	−9.63	0.79	−11.18	−8.08
	1999	−5.84	0.80	−7.39	−4.22
	2000	−3.11	0.69	−4.44	−1.74
	2001	−1.65	0.67	−2.98	−0.36
	2002	−0.45	0.68	−1.81	0.87
	2003	4.28	0.70	2.94	5.69
	2004	7.069	0.79	5.51	8.62
	2005	9.33	0.95	7.44	11.15
Girls	1998	−11.27	0.74	−12.73	−9.83
	1999	−7.79	0.71	−9.19	−6.41
	2000	−4.37	0.61	−5.59	−3.18
	2001	−1.00	0.72	−2.38	0.43
	2002	0.58	0.64	−0.67	1.85
	2003	4.07	0.68	2.71	5.38
	2004	8.07	0.70	6.68	9.45
	2005	11.71	0.85	10.07	13.40
